# Commentary: Cerebellar direct current stimulation enhances on-line motor skill acquisition through an effect on accuracy

**DOI:** 10.3389/fnhum.2015.00578

**Published:** 2015-10-20

**Authors:** Matthieu P. Boisgontier

**Affiliations:** Movement Control and Neuroplasticity Research Group, Group Biomedical Sciences, KU LeuvenLeuven, Belgium

**Keywords:** tDCS, sensory-motor integration, proprioception, motor control, skill acquisition

In the past decade, human cortical activity has been shown to be modulated by applying direct low electrical current. The current flowing through the skull and brain between two surface electrodes increases excitability of the cortical tissue under the anode and decreases it under the cathode. This transcranial direct current stimulation (tDCS) improves motor adaptation to environmental change (Galea et al., 2011; Hardwick and Celnik, 2014; Parikh and Cole, 2015) as well as skilled motor learning (Reis et al., 2009; Prichard et al., 2014; Cantarero et al., 2015). The cortical sites that have been shown to impact motor control under tDCS stimulation are the primary motor area (M1; Nitsche et al., 2003) and the cerebellum (Galea et al., 2011).

A recent study investigated the effects of cerebellar tDCS (ctDCS) on motor skill learning (Cantarero et al., 2015). Participants attempted to quickly and accurately navigate a cursor on a screen by modulating the isometric key pinch force. To perform the sequential visual isometric pinch task (Reis et al., 2009; Cantarero et al., 2015), the brain has to integrate three types of information: visual feedback, force feedback (proprioception), and motor command. Therefore, improving sensory-motor integration would improve performance in this task.

Recently, the cerebellum has shown multimodal arrangements, providing an anatomical basis for this sensory-motor integration. Proville et al. (2014) demonstrated that sensory and motor information from the cerebral cortex converges on single cells in the cerebellum. Proprioceptive information from the spinocerebellar tract and motor information from the cerebral cortex also converge on single cells in the cerebellum (Huang et al., 2013; Requarth et al., 2014). Therefore, cerebellar stimulation can potentially improve sensory-motor integration.

Results of Cantarero et al. (2015) showed that anodal ctDCS improved motor accuracy relative to anodal tDCS and sham groups. The improved accuracy was not associated with reduced movement speed (Cantarero et al., 2015, Figure 3C). This change in the speed-accuracy tradeoff demonstrated an improvement in motor skill. Although not reported in the text, Figure 3A in Cantarero et al. (2015) clearly showed that the effect of ctDCS on accuracy was not gradual but immediate (Day 1, Block 2), consistent with the immediate enhancement of conditioned eyeblink responses reported during anodal ctDCS (Zuchowski et al., 2014). After this immediate shift, accuracy remained stable across training days and did not show a typical learning curve.

The effect of ctDCS on motor accuracy is unlikely related to the visual system which can hardly be improved. This effect may rather result from improved proprioception. Specifically, in the sequential visual isometric pinch task, improving proprioception would improve the ability to accurately match visual and muscle-force information. Based on studies testing cerebellar patients (e.g., Bhanpuri et al., 2013), it has been suggested that the integration of peripheral proprioceptive information and central motor information in the cerebellum (Huang et al., 2013; Requarth et al., 2014) produces refined proprioception (Boisgontier and Swinnen, 2014). Therefore, if ctDCS improves proprioception this is likely through an improvement of this integration. Sensory-motor integration is performed on three types of information: Space, quantity, and time (Walsh, 2003). Proprioception refers to space (e.g., position of the segments) and quantity (e.g., intensity of the muscle contraction). Wessel et al. (2015) showed that anodal ctDCS does not significantly improve online motor skill learning in a synchronization tapping task, suggesting that anodal ctDCS does not impact the temporal component of sensory-motor integration.

M1 tDCS also immediately improves performance in a motor skill task, as demonstrated by Prichard et al. (2014), and visible in Reis et al. (2009). Nevertheless, the immediate effects for ctDCS and M1 tDCS may have different grounds. As described above, immediate ctDCS effects may result from an improved proprioception. Immediate M1 tDCS effects could instead be due to refined spatiotemporal patterns of neuronal activity in M1, which have been associated with improved motor control (Peters et al., 2014). Furthermore, Parikh and Cole (2015) showed that applying M1 tDCS during practice of the Grooved pegboard test improved performance on a grip-lift task, thereby suggesting that this tDCS-induced refinement of neuronal activity in M1 could be transferred between tasks.

In conclusion, Cantarero et al. (2015) study showed an immediate ctDCS effect on movement accuracy. Here I propose that this effect is mediated by improved sensory-motor integration in the cerebellum resulting in refined proprioception. This immediate effect has also been reported in motor skill learning studies using M1 tDCS, although the underlying mechanism here may instead be related to the refinement of the spatiotemporal patterns of neuronal activity in M1.

## Conflict of interest statement

The author declares that the research was conducted in the absence of any commercial or financial relationships that could be construed as a potential conflict of interest.
